# Effect of a multi-component palliative care intervention on goals of care discussions for critical patients in the emergency department

**DOI:** 10.1007/s11739-025-04048-5

**Published:** 2025-07-23

**Authors:** Julia Murray, Zacharia Grami, Katherine Benson, Christopher Hritz, Samantha Lawson, Corita Reilley Grudzen, Allison Cuthel, Lauren Talanda-Fath Southerland

**Affiliations:** 1 The Ohio State University College of Medicine, Columbus, OH USA; 2 Department of Emergency Medicine, The Ohio State University Wexner Medical Center, 750 Prior Hall, 376 W 10th Ave, Columbus, OH 43210 USA; 3 Division of Palliative Medicine, Department of Internal Medicine, The Ohio State University Wexner Medical Center, Columbus, OH USA; 4 Department of Medicine, Memorial Sloan Kettering Cancer Center, New York, NY USA; 5 Department of Palliative Medicine, NYU Langone Health, New York, NY USA

**Keywords:** Advance care planning, Palliative medicine, Electronic health records, Emergency department, Quality improvement, Healthcare power of attorney

## Abstract

Goals of care (GOC) discussions are vital to understanding patients’ values and preferences during serious illness, but they occur infrequently during Emergency Department (ED) care. We report a single site sub-study of a stepped wedge pragmatic trial of a multi-component intervention of primary palliative care in the ED (PRIM-ER), focused on GOC conversations. A retrospective cohort study of ED patients who either died or had a referral to hospice within 48 h or were admitted to an Intensive Care Unit (ICU). The PRIM-ER intervention consisted of: (1) evidence-based multidisciplinary education; (2) simulation-based workshops on serious illness communication; (3) clinical decision support; and (4) audit and feedback. The pre-intervention period was September 2018–August 2019 and the post-intervention period was November 2019–April 2020. Of the 153 included cases, 76 (49.7%) were pre-intervention and 77 (50.3%) were post-intervention. Only 6.5% (*n* = 10) had a Do Not Resuscitate order prior to the ED visit. The intervention did not change the proportion of patients receiving a GOC conversation in the ED (38.2% vs 40.3%, *χ*^2^ *p* = 0.79). Presenting with a cancer-related complaint (OR 12.9, 95% CI 4.15–39.92) or a respiratory emergency (OR 3.02, 95% CI 0.15–0.81) were associated with increased odds of a GOC conversation occurring. Of the 60 GOC conversations that occurred, 76.7% (*n* = 46) resulted in a change in code status, patient care plans, hospice, or updated advance care planning documents. While the intervention did not increase this secondary outcome of GOC conversations, the discussions that did occur frequently impacted ED care.

**Supplementary Information:**

The online version contains supplementary material available at 10.1007/s11739-025-04048-5.

## Background

In the US, 11% of deaths occur in Emergency Departments (ED) and 33% of those who die visited the ED in the month before death [1]. Although ED patients bear a high burden of critical and terminal illness and injuries, palliative care is rarely provided. This is possibly related to infrequent goals of care (GOC) conversations in the ED. 

GOC conversations involve building rapport discussing prognosis or delivering bad news, eliciting values, and shared medical decision making [2]. GOC conversations ensure that patients receive value-oriented care. GOC conversations and early referral to palliative care reduces intensive care unit (ICU) and hospital admissions and increases hospice care utilization [3]. Time constraints in the ED require that GOC conversations be rapid and focused on immediate goals [4]. Additional barriers include overcrowding, lack of training or comfort with advance care planning (ACP), lack of rapport or relationship with the patient, concerns about patient expectations, and role identity [5, 6]. Some ED physicians feel GOC conversations should be the oncologist or inpatient team’s workload/role [7]. Despite these limitations and barriers, GOC conversations are feasible in the ED, however, we do not know how frequently these conversations happen in current ED practice as it is difficult to abstract from electronic health records (EHRs) as they are often free text in inconsistent and unstructured locations [8, 9].

The PRIM-ER Study (NIH UH3AT009844) [10] was a 33-hospital pragmatic trial of a multi-component intervention involving training emergency nurses and physicians in palliative care techniques, clinical decision support, and audit and feedback. One disadvantage of large multicenter trials is that collating results from many sites can result in a lack of granularity on process factors or site-specific outcomes. Our site team wished to better investigate whether the PRIM-ER intervention changed physician behavior at our institution. We undertook a single-site sub study of the PRIM-ER study focused on whether the intervention increased the number of critically ill patients in the ED receiving a GOC conversation. The secondary outcomes were compliance with the EHR intervention, defined as use of a new GOC conversation note template, and outcomes of GOC conversations. These process outcomes are important as they describe the level of adoption and provide insight for future interventions.

## Methods

This study was a quality improvement initiative as part of a larger multicenter stepped wedge pragmatic trial [10]. The sub-study is a pre/post cohort study of ED patients at a large academic tertiary care hospital in the Midwest United States with 80,000 ED patient visits a year. Standards for quality improvement reporting excellence (SQuIRE) were followed [11]. Ethical approval for the primary study was granted by the New York University Grossman School of Medicine Institutional Review Board (ID: i18-00607) with a waiver of consent. This single-site study was approved by the institution’s review board (#2020H0004). Publication of data was withheld until the final parent study and data analysis were completed in 2024 to avoid potentially biasing other study sites or the analysis team. The primary study results are now available [12].

### Intervention

The site implementation team included an ED physician, nurse educators, palliative medicine physicians, and management support from the central trial team. The educational component included a 4-h simulation workshop in end-of-life communication (Education in Palliative and End-of-Life Emergency Medicine (EPEC-EM)) for faculty and advanced practice providers and one hour of online didactic training (End of Life Nursing Education Consortium Critical Care (ELNEC) for ED nurses. Education was completed by 79% of ED clinicians (*n* = 62/78) and 67% (*n* = 98/134) of the ED nurses. Educational emails and reminders were sent during the implementation month (September 2019). Staff joining the department after September 2019 received brief training on EHR changes and templates but did not undergo EPEC-EM or ELNEC.

**Fig. 1:**
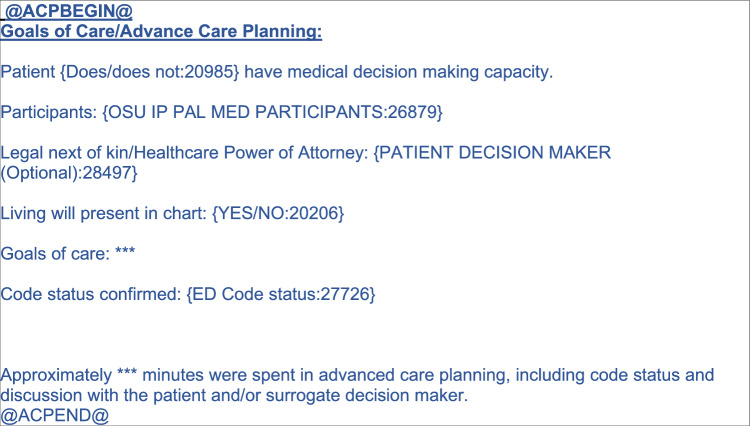
Template for ACP discussion documentation can be modified by the user with every use. The text bracketed by @ symbols are sectioning smart links that list this text in the Epic’s ACP activity. This allows future providers to easily find the information, instead of searching through the text of multiple notes. The drop-down menus in brackets allow for quick, guided documentation. For example, for participants, the drop-down allows the provider to easily pick from family, daughter, spouse, HPOA, chaplain, social worker and patient, among others. The sections with three asterixis (***) are easy to tab to and then add free text information

For the clinical decision support component of the intervention, a new GOC discussion documentation template was developed (Fig. 1) to standardize documentation and correspond to billing needs for the Centers for Medicare and Medicaid 99497 code for ACP conversations [13]. This included a more prominent link to the patient’s code status and advance care planning documents in an Epic navigator tab [8]. The intervention team also investigated and troubleshooted advance care planning documentation in the EHR [8]. No palliative care needs screening or alerts were implemented.

### Chart selection and power

The patients were identified by EHR query (Epic, Epic Systems Corporation, Verona, Washington) for ED encounters September 2018-April 2020 that met any of 3 criteria: (1) a referral order to hospice within 48 h of ED arrival, (2) death within 48 h of ED arrival, or (3) ICU admission from ED. These criteria were chosen to identify patients with critical illness that may have warranted GOC discussion during their ED visit. As few patients qualified under criteria 1, all were included. Many encounters were potentially qualifying under criteria 2 and 3 and so these encounters were randomized within each criteria group for study selection. An interim check was done to evaluate the group composition and equalize the proportions from each criteria group in the pre and post cohorts. The charts were excluded if there was no ED encounter, incomplete documentation, had an encounter date during the time of the intervention (September or October 2019), or if the ICU admission was not immediately from the ED encounter (for example, admitted to a regular ward for two days and later upgraded to ICU admission).

Based on a prior study of trauma patients at our site, we assumed a pre-cohort GOC conversation rate of 15% [14]. If the intervention increased the rate to 45%, we would need 47 patients per group to be 90% powered to detect this difference with an alpha of 0.05. As we expected variability due to the 3 types of inclusion criteria, the sampling goal was 75 charts per cohort for at least 150 participants.

### Chart abstraction

Chart abstraction was completed by trained study abstractors (medical students, residents, faculty) using REDCap, a HIPAA compliant research database [15]. Blinding to study group was impossible as the date of ED encounter was necessary to abstract the correct information. To avoid bias, chart abstractors were not funded by or involved with the parent PRIM-ER study, and all data collection was completed and analyzed prior to the final PRIM-ER findings. An abstraction codebook was developed during training and refined during chart review (see Supplemental Data A). All ED notes from the index encounter, including from physicians, nurses, advanced practice providers, social workers, and consultants were reviewed. The team preselected data elements at higher risk of misclassification for dual abstraction: type of emergency, presence of life-limiting illnesses, presence of a GOC conversation, and reason for the GOC conversation. The discrepancies were adjudicated by an independent third abstractor and inter-rater reliability was calculated.

### Data elements

Data elements included demographics, any life-limiting medical condition(s), type of emergency, initial and final code status, level of care provided (ICU, intermediate, or floor), ED disposition, and documentation of a GOC discussion. GOC conversation was defined as any of: (1) documentation of a discussion with a patient or their decision maker(s) regarding code status, advance care planning decisions or documents, or (2) a note containing one or more of the following terms (do not resuscitate, cardiopulmonary resuscitation (CPR), advance directive, power or attorney, Do Not Resuscitate (DNR), code status, advance directive, living will, POLST, goals of care, advance care planning), or (3) a positive search for documents containing advance directive, DNR, or hospice, or a specifically labeled “Advance Care Planning” note occurring during the ED stay. There had to be evidence of a conversation or discussion—routine note templates saying “Code status: Full” did not count as a documented GOC conversation. Key elements of a GOC conversation (decision-making capacity of the patient, legal next of kin, participants, presence of ACP documents, and code status) were noted if present. As a data check, the abstractor copied the specific text from the chart that defined “GOC conversation”. The reasons for GOC conversations were an actively dying patient, a newly diagnosed life-limiting illness, admission to the ICU, need for a procedure, or patient or family initiated.

Type of emergency was defined as the medical event warranting a visit to the ED, categorized as sepsis, trauma, stroke, other neurologic disease, cancer complications, COVID-19, heart failure, end stage renal disease, respiratory disease, cardiac arrest, or other. The life-limiting conditions present in the ED were categorized as oxygen-dependent lung disease, congestive heart failure, end-stage cancer, end-stage renal disease, stroke, severe injury, or other life-limiting conditions. More than one could be present.

Code status in Ohio is divided into Full Code (proceed with all disease directed therapies), Do Not Resuscitate–Comfort Care Arrest (DNRCC-A, do not resuscitate in the case of cardiac arrest), and Do Not Resuscitate–Comfort Care (DNRCC, comfort care measures only) [16].

### Analysis

Descriptive analysis of the patient population is reported as means and standard deviation and proportions with 95% confidence intervals as appropriate. The pre-and post-cohorts were compared using chi^2^ tests for proportions. Univariate logistic regression was used to look for factors associated with GOC conversation. The variables were pre-selected by the team and included age, gender, cohort, and type of emergency. The missing data were reported as missing.

## Outcomes

### Cohort derivation and data abstraction

The charts were randomly selected from 5953 potential ED encounters (22 with hospice referral, 92 deaths, and 5839 ICU admissions). We reviewed 197 and 44 were excluded (one duplicate encounter, 15 for ED visit time during the rollout, and 28 who were not ICU admissions). This resulted in 153 patients meeting inclusion criteria. Inter-rater reliability was excellent with agreement on whether a GOC conversation was documented at 96.7%, kappa 0.93. Agreement on the type of emergency was 93.7% with kappa 0.72.

### Characteristics of study patients

The total cohort (*n* = 153) included 14% (*n* = 21) who met the referral to hospice criteria, 41% (*n* = 63) who died within 48 h and 53% (*n* = 81) who were admitted to the ICU. The mean age of patients included in the study was 62.2 ± 16.5 years (range 19–103) and 44.8% were female. Five patients required a language interpreter. Gender, race, ethnicity, and interpreter use did not differ significantly between cohorts.

The cohorts had similar rates of underlying life-limiting illnesses (Table 1). There were 36 patients who did not have any documented underlying life limiting illness present (24 pre-cohort, and 12 post-cohort). Most presented in cardiac arrest, which limited the available medical history. Cohorts differed slightly in the type of presenting emergencies, with cardiac arrest in the pre-cohort (39% vs 13%, Table 2). The two cohorts also differed in that the pre-cohort timeframe was pre-COVID pandemic. The post-cohort include the first few months of the pandemic (March and April 2020). At this time, COVID testing was not readily available and the ED volumes were very low due to lockdown.

**Table 1:** Comparison of demographics and life-limiting illnesses present in the cohort of Emergency Department patients

	Pre-cohort (*n* = 76)		Post-cohort (*n* = 77)		Total (*n* = 153)		
	*n*	%	*n*	%	*n*	%	*p* value
Age (years) (mean, std dev)	61.9	± 15.5	62.4	± 17.6	62.2	± 16.5	0.86
Sex (female)	33	43.4%	36	46.7%	69	45.1%	0.68
Oxygen dependent lung disease	8	11%	6	8%	14	9%	0.56
Congestive heart failure	14	18%	14	18%	28	18%	0.97
End-stage renal disease	4	5%	4	5%	8	5%	0.99
Cancer, not in remission	11	14%	17	22%	28	18%	0.22
Severe infection	15	20%	19	25%	34	22%	0.46
Stroke	5	7%	11	14%	16	10%	0.12
Severe injury	6	8%	11	14%	17	11%	0.21
Other life limiting illnesses	51	67%	43	56%	94	61%	0.15

Chi^2^ *p* values are presented only to highlight differences between the cohorts. Student *t*-test used to compare age

**Table 2:** Types of presenting emergencies in ED patients with critical illness before and after an education intervention for goals of care discussions

	Pre-cohort (*n* = 76)		Post-cohort (*n* = 77)		Total (*n* = 153)		*p* value
Sepsis	10	13%	11	14%	21	14%	0.84
Trauma	7	9%	9	12%	16	10%	0.62
Stroke	5	7%	10	13%	15	10%	0.18
Other neurologic disease	6	8%	4	5%	10	7%	0.5
Cancer-related	12	16%	14	18%	26	17%	0.7
COVID-19	0	0%	1	1%	1	1%	0.32
CHF	5	7%	7	9%	12	8%	0.56
ESRD	1	1%	1	1%	2	1%	0.99
Respiratory disease	13	17%	13	17%	26	17%	0.97
Cardiac arrest	30	39%	10	13%	40	26%	< 0.01
Other emergencies	14	18%	25	32%	39	25%	0.05

All numbers listed as number, %. Chi^2^ *p* values are presented only to highlight differences between the cohorts

### Primary outcome: goals of care discussions

The intervention did not change the proportion of patients receiving a GOC conversation in the ED (38.2% pre-cohort vs 40.2% post-cohort, *χ*^2^ *p* = 0.79). The GOC conversations were documented by the ED physician team (*n* = 42, 63%) and/or the ED case management/social work team (*n* = 35, 43%), with six conversations only documented by a consultant. Most common reasons for GOC conversations were an actively dying patient (43%, *n* = 29), a newly diagnosed life-limiting illness (47%, *n* = 28), and/or admission to the ICU or need for a procedure (40%, n = 24). A fifth (n = 13, 22%) were initiated by patient or family request. Changes from initial code status on ED arrival to final code status are reported in Table 3. There was no difference in final code status between the cohorts (chi^2^ test, *p* = 0.36).

**Table 3:** Code status of critically ill patients in the Emergency Department upon arrival and at the end of their ED visit

Code status	Initial (*n*, %)		Final (*n*, %)	
Full code	78	51%	64	41.8%
DNR-CC: do not resuscitate–comfort care	2	1.3%	21	13.7%
DNR-CCA: do not resuscitate–comfort care in case of arrest	8	5.2%	12	7.8%
Not Documented	65	42.5%	56	36.6%

Of the 60 GOC conversations that occurred, 76.7% (*n* = 56) were impactful, as 52% (*n* = 31) resulted in a change in code status, 53% (*n* = 32) resulted in a change in patient care, and 20% (*n* = 12) resulted in updated advance care planning documents. The intervention increased billing statements for GOC (0 pre-cohort vs 7 post-cohort) but did not change the mean number of key documentation elements (1.2 vs 1.3 elements (*p* = 0.9)). Most documented elements were the participants (87% of GOC conversations) and patient’s code status (80% of GOC conversations).

On univariate analysis, presentation for complications of cancer (OR 12.9) or a respiratory emergency (OR 3.02) significantly increased the odds of having a GOC conversation (Table 4). Patients presenting with trauma (OR 0.09) or cardiac arrest (OR 0.35) had lower odds. All 10 patients with an initial code status that was not full code (DNR-CC or DNR-CCA) had a GOC conversation.

**Table 4:** Univariate analysis of a patient’s age, gender, and type of emergency and the odds of a goals of care conversation occurring in the Emergency Department among a sample of 153 patients

	OR	*p*	95% CI
Age	1.03	< 0.01	1.01–1.06
Gender (reference male)			
Female	1.38	0.33	0.072–2.66
Type of presenting emergency			
Sepsis	1.04	0.92	0.46–2.35
Trauma	0.09	0.02	0.11–0.69
Stroke	1.4	0.535	0.48–4.09
Cancer-related	12.9	< 0.01	4.15–39.92
Congestive heart failure	1.12	0.856	0.33–3.70
Respiratory disease	3.02	0.013	1.26–7.20
Cardiac arrest	0.35	0.014	0.15–0.81

The emergencies of COVID-19 and end stage renal disease were not included due to too few encounters in the dataset

## Conclusions/Lessons learned

This is a single site analysis of an intensive multi-component intervention to integrate palliative medicine principles into emergency care. Despite education and reminders, we did not see GOC conversations for critically ill patients increase. While the intervention did not have an effect on hospital admission or subsequent health care use in the larger cluster randomized clinical trial, ascertainment of the presence of GOC was outside the scope of the larger study [12]. Thus, we cannot generalize the results of this single site to the larger trial. However, the lack of change in physician behavior at this site likely impacted the study results from this site.

While we did not increase GOC conversations, this study reaffirms that these conversations are impactful. As only 40% of critically ill patients in the ED received a GOC conversation and a third were still missing a code status order upon disposition, we have significant room for improvement. Prior interventions to increase GOC documentation in other hospital settings have also met with limited adoption. For example, a project to fill out a GOC discussion form for hospitalized patients reached only 63% compliance [17]. Another investigation of the use of structured ACP fields found only 43% compliance and almost a fourth of the data was incorrect [18]. In the large Kaiser Permanente healthsystem, a rollout of a care directives tab in the EHR (similar to the EHR changes for this study) did not change documentation of GOC [19]. Improving quality and quantity of ED GOC conversations is an important but lofty goal, and while education and EHR improvements are necessary, these interventions seem insufficient to change provider behavior [20].

Our data also contribute to known barriers/facilitators to GOC conversations in the ED [21]. A cancer diagnosis was associated with greater odds of a GOC conversation, similar to the findings of a prior study of general medical hospital patients [22]. It is possible that oncology patients had a definite progression or disease complication that allowed the ED provider to feel more comfortable prognosticating. In contrast, patients in the ED for traumatic injuries had far lower odds of a GOC conversation. Improving the rate of early GOC conversations for injured older patients is a new focus from national trauma quality programs [23, 24]. The parent study and this study were also not focused on patients in cardiac arrest, which is a population that has different goals of care conversation structures and palliative care needs [25–27].

Our final finding is the value of multidisciplinary teams in ED conversations. The case management/social work team was involved in almost half of GOC discussions. Training ED social workers and case managers to lead these conversations may take some of the burden off busy emergency medicine physicians [28–30]. Future interventions should also include training for residents and trainees. The impact at this academic site was likely diluted as resident physicians are involved in most ED patient care but were not included in the educational roll out. We had initially planned to provide EPEC-EM training to emergency medicine residents in small groups throughout 2020, however, the COVID-19 pandemic eliminated in-person education.

The limitations include small sample size and single site nature of this study. Additionally, GOC conversations were not an outcome of the primary study but rather an internal measure decided on by our site team as a proxy for the impact of the educational component and a measure of care quality. The changes in ED population, volumes, and resources during March and April 2020 due to the COVID pandemic and lockdowns could also have impacted the post-cohort population. At this time, our ED had low volumes and was not in a COVID surge. The low number of COVID positive patients could also be due to the lack of testing. Finally, we evaluated clinical documentation only. The lack of a documented GOC conversation does not mean that this was never discussed. Far more is done for patients than what is documented.

In conclusion, a multi-component intervention did not increase GOC conversations in the ED. We identified different patient factors which were associated with the odds of a GOC conversation occurring and potential target populations for future interventions.

## Supplementary Information

Below is the link to the electronic supplementary material.

Supplementary file1 (XLSX 18 KB)

## Data Availability

Research data is available upon request. Please contact the corresponding author.
